# Promoting the well-being of rural elderly people for longevity among different birth generations: A healthy lifestyle perspective

**DOI:** 10.3389/fpubh.2023.1050789

**Published:** 2023-02-23

**Authors:** Xian Li, Min Gao, Meijie Chu, Shiling Huang, Zhiwei Fang, Tianmu Chen, Chun-Yang Lee, Yi-Chen Chiang

**Affiliations:** ^1^State Key Laboratory of Molecular Vaccinology and Molecular Diagnostics, School of Public Health, Xiamen University, Xiamen, China; ^2^School of International Business, Xiamen University Tan Kah Kee College, Zhangzhou, China

**Keywords:** longevity, subjective wellbeing, lifestyle, cohort study, rural elderly

## Abstract

**Background:**

Wellbeing may have a protective role in health maintenance. However, no specific study clarified the particular protective effect of the subjective wellbeing of rural elderly people on survival probability. Few studies have examined the effect of the lifestyle of rural elderly people on their subjective wellbeing from different perspectives. We investigated whether improving subjective wellbeing increased the probability of longevity of rural elderly people and the effects of lifestyle behaviors on the subjective wellbeing of rural elderly people in different birth generations.

**Materials and methods:**

Data were derived from the China Health and Nutrition Survey (CHNS), which is an ongoing open cohort study that adopts a multistage, random clustered sampling process. We used the data of elderly people who were aged 65 or over during 2006–2015 for analysis. The Kaplan–Meier method and log-rank test found that the survival probability of rural elderly people was significantly lower than urban elderly people. Based on a sample of rural elderly people, Cox regression and generalized estimating equations were performed as further analyses.

**Results:**

A total of 892 rural elderly people aged 65 or over were included in the sample in 2006. High subjective wellbeing was a protective factor against death. The subjective wellbeing of rural elderly people born in the 1940s/1930s/1908–1920s birth generations first decreased then increased. For rural elderly people born in the 1940s, there were significant positive effects of a preference for eating vegetables and walking/Tai Chi on subjective wellbeing. For rural elderly people born in the 1930s, preferences for eating vegetables, reading, and watching TV all had significant positive effects on subjective wellbeing. Rural elderly people born in the 1908–1920s who preferred watching TV had more subjective wellbeing.

**Conclusion:**

Improving subjective wellbeing extended the life span and reduced mortality risk in rural elderly people and may be achieved by the shaping of a healthy lifestyle, such as preferences for eating vegetables, walking/Tai Chi, and reading.

## 1. Introduction

The world's population is aging, and most countries are experiencing growth in the number and proportion of the elderly population (1). Approximately 9% of people worldwide were over 65 years old in 2021 (2). There are 190 million citizens over 65 years of age in China, which accounts for 13.5% of the total population (3). Active and healthy aging is the theme of the era. The English Longitudinal Study of Aging documented participants' experience of growing old in the context of active aging in the twenty-first century (4). Older adults are increasingly seen as contributors to development (5). Notably, China has the largest elderly population in the world (6), and the proportion of elderly Chinese aged 65 years and over in rural areas is 17.72%, which is 6.61% higher than urban areas (7). The average life expectancy of rural elderly people aged 65 and over is 16.69 ± 0.25 years, which is lower than the 18.33 ± 0.35 years of the urban elderly aged 65 and over (8). Rural revitalization pushes country prosperity forward. Improving the life expectancy of elderly people has become a key goal of the full life cycle of elderly care services. Chinese rural elderly people tend to report higher negative emotions than urban elderly people (9). Helping rural elderly people realize their pursuit of a better and happy life is a major concern of Chinese society.

Wellbeing may be good for the mind and the body (10). Higher subjective wellbeing is associated with good health (11). A longitudinal study demonstrated a significant association between subjective wellbeing and longevity (12–14), and eudemonic wellbeing increased survival (15). Several factors, such as educational level and lifestyle, significantly influenced the overall subjective wellbeing of older adults (16). A healthy lifestyle markedly increases life span and improves individuals' subjective wellbeing (17, 18). Lifestyle behaviors include physical activity, healthy diet, sleep, smoking, and drinking (19). Eating fruit and vegetable had positive effects on subjective wellbeing (20, 21). Physical activity was a protective factor for subjective wellbeing in older adults (22), and it reduced their risk of late-life depression (23). Many older adults would choose to make some changes to increase their wellbeing in their later life (24).

Life evaluation and wellbeing are relevant to health and quality of life as people age (15). Due to differences in the background of the times, age and birth generation are related to the prevalence and changes in lifestyle behavior (25). The present study compared the differences in life span between urban and rural elderly people based on an ongoing open cohort, the China Health and Nutrition Survey (CHNS). We tested two hypotheses. First, we examined whether improving subjective wellbeing increased the probability of longevity of rural elderly people. Second, we investigated the effects of lifestyle behaviors on the subjective wellbeing of rural elderly people of different birth generations.

## 2. Materials and methods

### 2.1. Participants and survey procedures

Data were derived from the CHNS, which is an ongoing open cohort study that adopts a multistage, random clustered sampling process. First, we chose nine provinces that varied substantially in geography, economic development, public resources, and health indicators. Second, two cities (one large and one small) and four counties (stratified by income, one high, one low and two middle income) were randomly selected per province. Third, two urban and two suburban communities were randomly selected within cities, and one community and three rural villages were randomly selected within counties. Finally, 20 households per community or village were randomly selected for participation. CHNS was approved by the Institutional Review Boards of the University of North Carolina at Chapel Hill and the Chinese Center for Disease Control and Prevention. All participants provided written informed consent for their participation in the survey.

**Figure 1:**
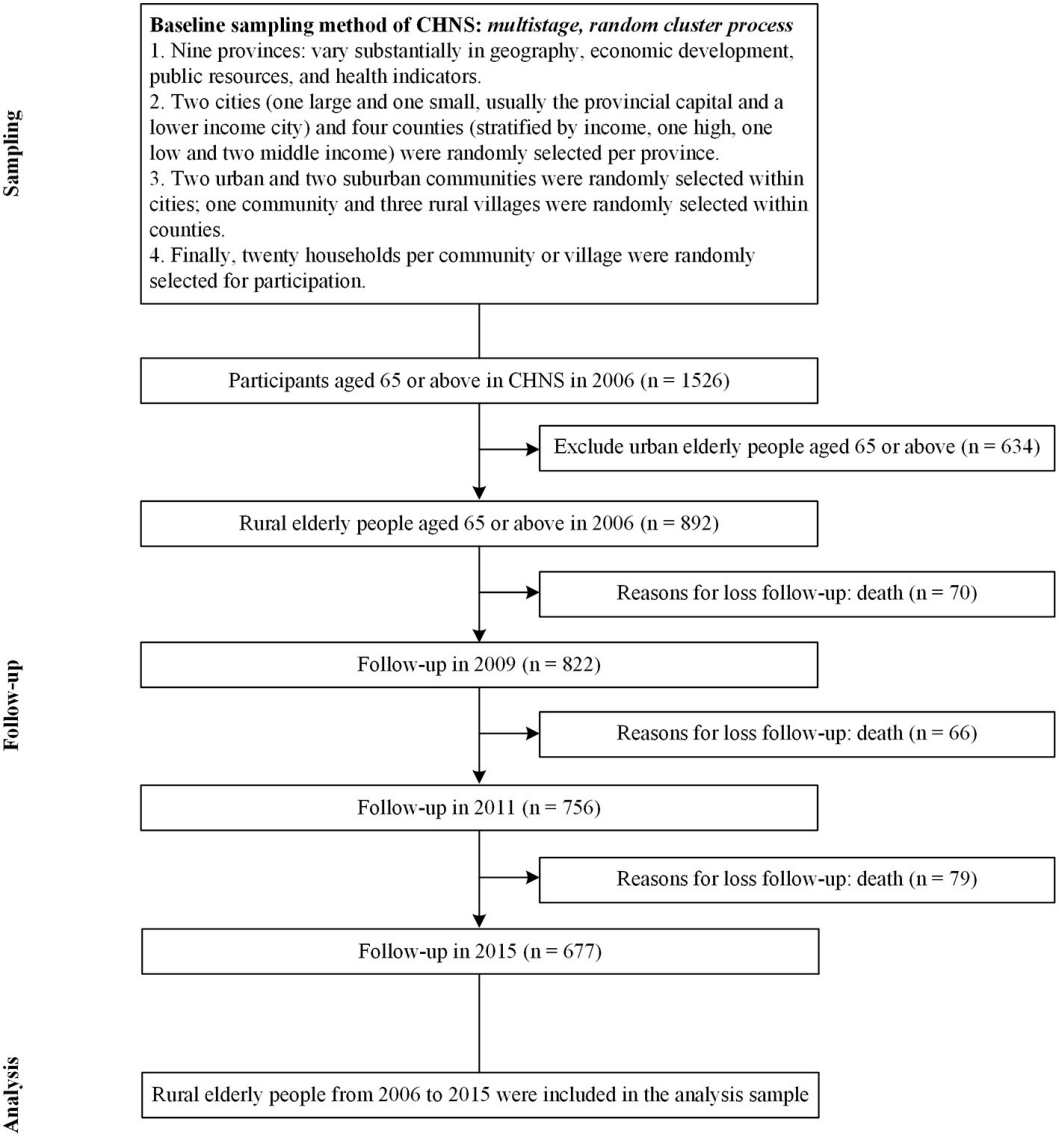
Flowchart.

**Figure 2:**
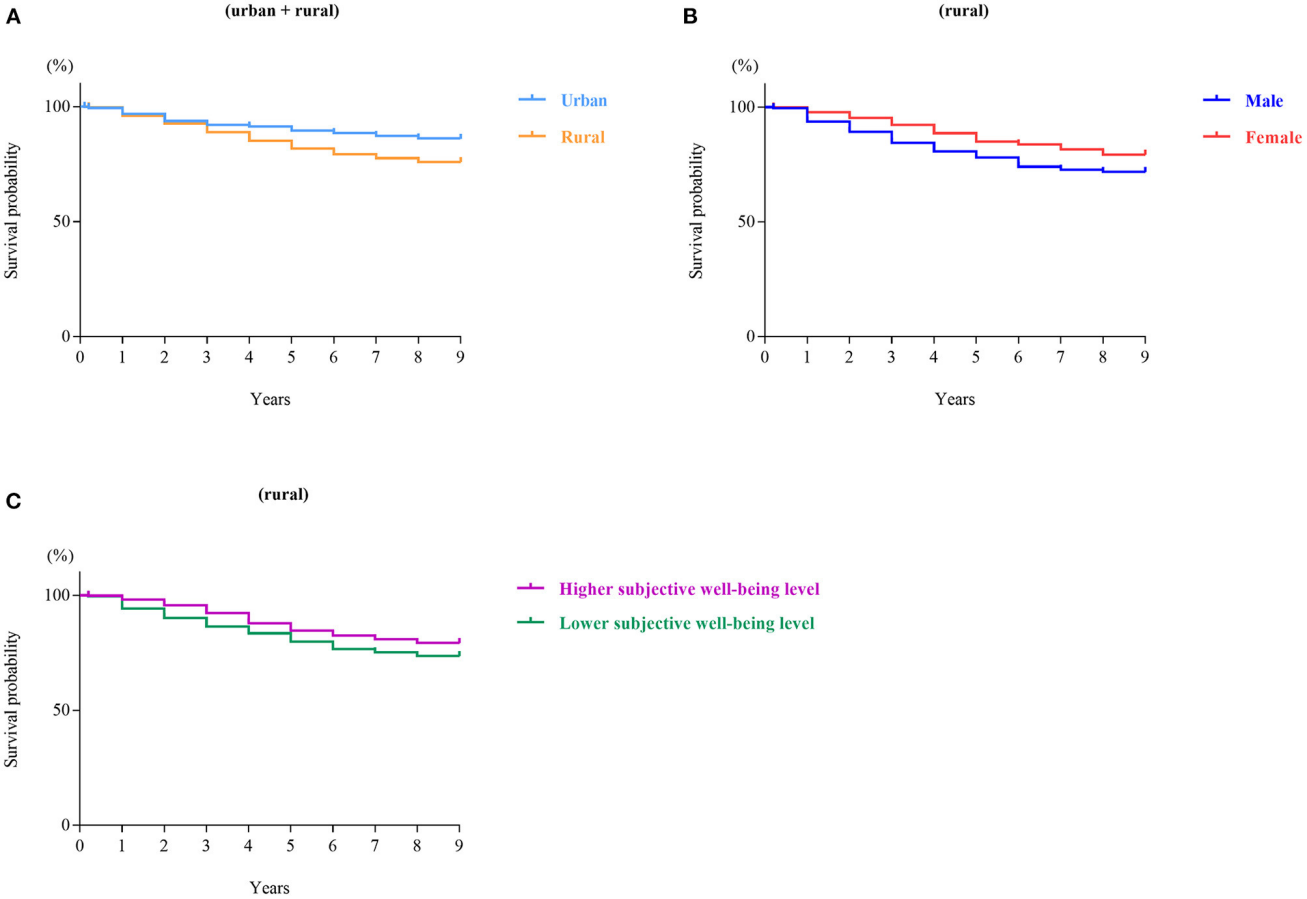
Survival curves among Chinese elderly people in urban/rural areas. **(A)** Urban + rural, **(B)** rural, **(C)** rural.

This study used the CHNS data from 2006, 2009, 2011 and 2015. As shown in Figure 1, of the 1526 participants aged 65 or over in 2006, 892 lived in rural areas, and 634 lived in urban areas. Comparisons of the survival probability of the rural and urban elderly in 9-year follow-up (Figure 2A) found that the survival probability of rural elderly people was significantly lower than urban elderly (*p* < 0.0001). This study focused on a sample of rural elderly people who were followed up from 2006 to 2015, with a follow-up rate of 75.9%. The reason for loss to follow-up was death.

### 2.2. Measurements

#### 2.2.1. Outcome variable

Subjective wellbeing refers to how people experience and evaluate their lives and specific domains and activities in their lives (26), which include emotional experiences and cognitive evaluations (life satisfaction). It was measured using four items: (i) How do you rate the quality of your life at present? (ii) I have as much pep as last year? (iii) I am as happy now as when younger? (iv) As I get older, things are better than I thought they would be. Each item was scored on five-point scale. After adjusting the reverse scoring, the total score was 4 to 20. Higher scores indicate higher subjective wellbeing. The Cronbach's α was 0.67–0.76 from 2006 to 2015, which indicated moderate reliability.

#### 2.2.2. Lifestyles

Lifestyles included food and activity preferences, and items were rated on a five-point scale ranging from “dislike very much” to “like very much.”

*Food preferences* were measured by asking participants “How much do you like this food: (i) fruits; (ii) vegetables; (iii) soft drinks and sugared fruit drinks?”

*Activity preferences* were measured by asking participants “How much do you like to participate in this activity: (i) walking, Tai Chi; (ii) body building; (iii) sports (ping pong, badminton, tennis, soccer, basketball, volleyball); (iv) reading; (v) watching TV; (vi) playing computer/video games, surfing the internet?” The first three activities are physical activities, and the last three activities are sedentary activities.

### 2.3. Statistical analysis

Descriptive statistics are expressed as frequency and percentages and means and standard deviation. We estimated and graphed survival curves using the Kaplan–Meier (KM) method, which involves computing the probabilities of occurrence of events at a certain point in time (27), and the log-rank test was used to compare two survival curves. After validation of proportional hazards assumption, Cox regression was used to examine the relationship between subjective wellbeing and survival outcome. This relationship was further tested using “mean ± SD” of subjective wellbeing as a cutoff, which helped determine their causal relationship. Cox's semiparametric model is widely used in the analysis of survival data to explain the effect of explanatory variables on hazard rates (28). Generalized estimating equations (GEE) were performed to clarify whether food preferences and activity preferences influenced the subjective wellbeing of Chinese rural elderly people across different birth generations after accounting for sex and highest educational level. During the follow-up period, there were no missing demographic variables, such as age, sex, or highest educational level, for surviving rural elderly people, except for subjects who died. For missing lifestyle and outcome variables for survivors, we used the answers of the previous session among the surviving rural elderly people to replace the missing value of the same variables/items. Statistical analyses were performed using SAS 9.4. GraphPad Prism 7.00 and Microsoft Visio 2019 program were used to draw figures.

## 3. Results

Descriptive statistics of the sample from 2006 to 2015 are shown in Table 1. A total of 892 rural elderly people aged 65 or over were included in the sample: 153 people were born in the 1940s, 542 people were born in the 1930s, and 197 people were born in the 1908–1920s. More than half of the subjects were female, and males accounted for 44.84% of the sample. Of the 892 rural elderly people, 63.23% never attended school, 19.96% graduated from primary school, and 9.08% had a lower middle school degree. Due to deaths, there were 677 rural elderly people in 2015 after 4 rounds of follow-up. The average score of subjective wellbeing in the 4 rounds of the survey fluctuated between 11.92 and 12.47.

**Table 1:** Descriptive statistics of the follow-up sample.

**Variables**	**2006 (*****n*** = **892)**		**2009 (*****n*** = **822)**		**2011 (*****n*** = **756)**		**2015 (*****n*** = **677)**	
	***n***	**(%)**	***n***	**(%)**	***n***	**(%)**	***n***	**(%)**
**Birth generations**								
1940s	153	(17.15)	147	(17.88)	141	(18.65)	133	(19.65)
1930s	542	(60.76)	509	(61.92)	474	(62.70)	425	(62.78)
1908–1920s	197	(22.09)	166	(20.19)	141	(18.65)	119	(17.58)
**Sex**								
Male	400	(44.84)	357	(43.43)	322	(42.59)	287	(42.39)
Female	492	(55.16)	465	(56.57)	434	(57.41)	390	(57.61)
**Highest educational level**								
None	564	(63.23)	564	(68.61)	511	(67.59)	463	(68.39)
Graduated from primary school	178	(19.96)	131	(15.94)	125	(16.53)	109	(16.10)
Lower middle school degree	81	(9.08)	71	(8.64)	67	(8.86)	57	(8.42)
Upper middle school degree	25	(2.80)	23	(2.80)	23	(3.04)	21	(3.10)
Technical or vocational degree	34	(3.81)	24	(2.92)	18	(2.38)	21	(3.10)
University or college degree	10	(1.12)	9	(1.09)	12	(1.59)	6	(0.89)
Master's degree or higher	0	(0.00)	0	(0.00)	0	(0.00)	0	(0.00)
	**Mean**	**(SD)**	**Mean**	**(SD)**	**Mean**	**(SD)**	**Mean**	**(SD)**
Subjective wellbeing	12.47	(2.74)	11.97	(2.76)	11.92	(2.80)	12.03	(2.81)

To evaluate whether sex and subjective wellbeing were associated with longevity in Chinese rural elderly people, Kaplan–Meier survival curves were generated using the 2006 data. As shown in Figure 2B, females lived longer than males (*p* = 0.0046). Figure 2C indicates that rural elderly people whose subjective wellbeing was higher than or equal to the average lived longer than rural elderly people whose subjective wellbeing was lower than the average (*p* = 0.0368). For the CHNS from 2006-2015, the categorical variables, which included sex and the bicategorical subjective wellbeing level with the mean plus standard deviation as a cutoff, were verified using the Kaplan-Meier survival curve method and the cumulative hazard function method, respectively, and both sets of curves for both variables were parallel and non-crossed. The influence of subjective wellbeing of continuous variables on survival outcomes was examined using the Schoenfeld residual method. The residuals fluctuated around 0, and there was no obvious change trend with the increase of time rank. The Pearson correlation coefficient between the Schoenfeld residual and time rank was not significant (*p* = 0.221). In summary, these data satisfied the proportional hazards assumption that the impact of covariates on survival possibility did not change over time, and the data are appropriate for a Cox regression analysis. After controlling for sex, Cox regression analysis suggested that high subjective wellbeing was a protective factor against death (HR, 0.90; 95% CI, 0.82–0.99), with a 10% lower risk of death when subjective wellbeing was higher than or equal to the average level. When subjective wellbeing was higher than or equal to the sum of the mean and standard deviation, there was a 69% lower risk of death (HR, 0.31; 95% CI, 0.11–0.85).

**Figure 3:**
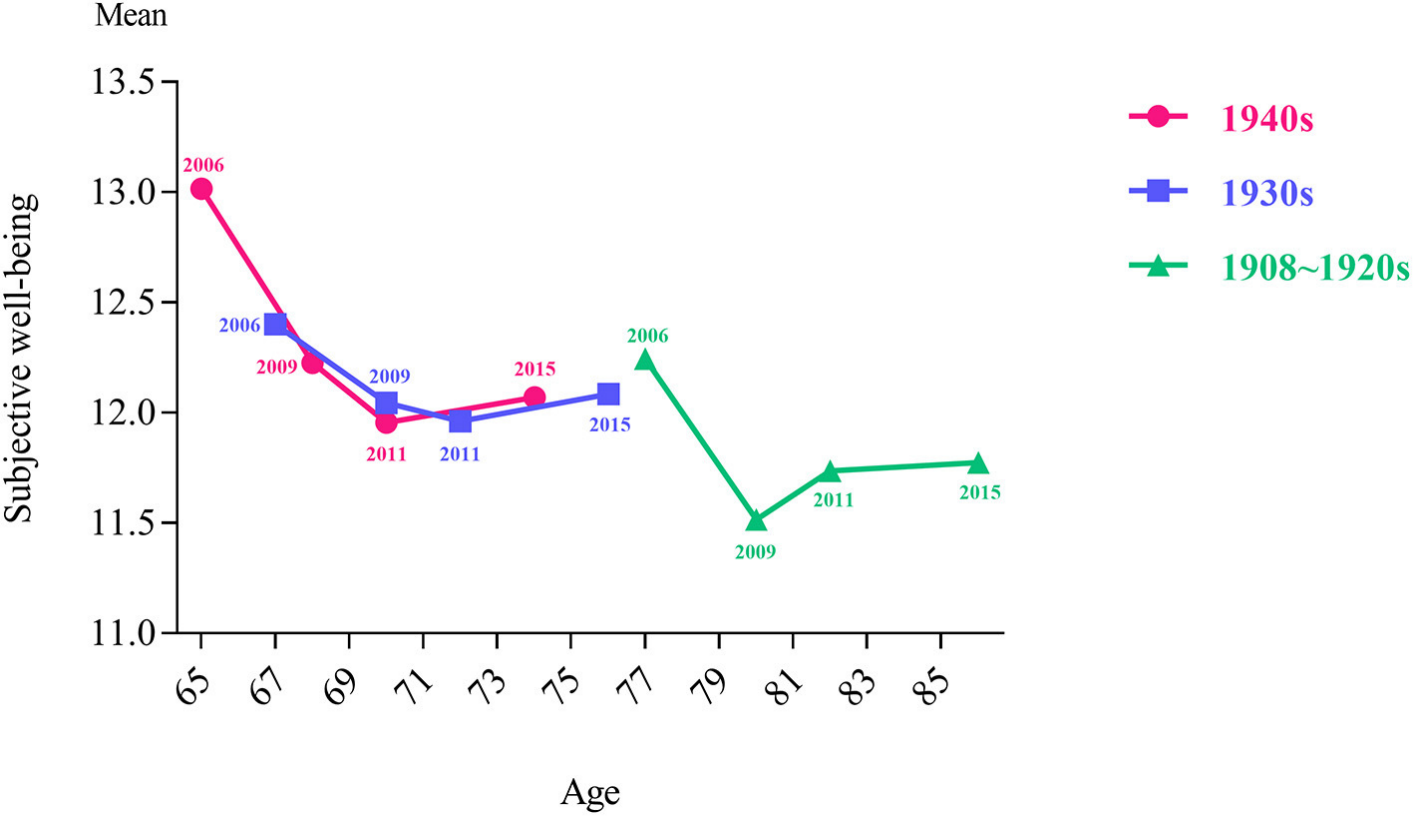
Subjective wellbeing level of Chinese rural elderly people in different birth generations during 2006–2015.

To further examine the trajectories in subjective wellbeing of rural elderly people in the four-wave survey by different birth generations, as shown in Figure 3, the horizontal axis corresponding to each birth generation group data point represents the minimum age of the group in 2006. The results showed that the subjective wellbeing of rural elderly people in the three groups of birth generations (1940s, 1930s, and 1908–1920s) all decreased then increased slightly. In the same round of surveys, the subjective wellbeing was lower for rural elderly people with an earlier birth generation than for rural elderly people with a later birth generation. This result suggests that the characteristics of birth background affect happiness.

Based on the above findings, we used GEE to analyze the factors that affected the subjective wellbeing of rural elderly people by different birth generations after controlling for sex and highest educational level (Table 2). Model 1 revealed that there was a significant positive correlation between a preference for eating vegetables and subjective wellbeing (β = 0.874, *p* < 0.05) among rural elderly people born in the 1940s. They had a greater preference for walking or Tai Chi and a higher subjective wellbeing (β = 0.395, *p* < 0.05). Model 2 discovered that a preference for eating vegetables (β = 0.548, *p* < 0.05), preference for reading (β = 0.433, *p* < 0.01), and preference for watching TV (β = 0.416, *p* < 0.001) were all significantly positively associated with subjective wellbeing for rural elderly people born in the 1930s. For rural elderly people born in the 1908–1920s, Model 3-1 showed that a greater preference for watching TV was associated with a higher subjective wellbeing (β = 0.519, *p* < 0.05). We examined whether there was an interaction between the reading preference and educational level of rural elderly people of different birth generations. The results indicated that only rural elderly people born in the 1908–1920s had this interaction. Model 3-2 showed that a stronger reading preference and higher educational level were associated with higher subjective wellbeing (β = 0.374, *p* < 0.05).

**Table 2:** GEE analysis of subjective wellbeing of Chinese rural elderly people by different birth generation during 2006–2015.

**Variables**	**1940s**		**1930s**		**1908–1920s**			
	**Model 1**		**Model 2**		**Model 3-1**		**Model 3-2**	
	**β**	***p***	**β**	***p***	**β**	***p***	**β**	***p***
Intercept	8.428		8.004		8.778		9.060	
**Food preferences**								
Fruits	−0.204		0.087		−0.070		−0.091	
Vegetables	0.874	^*^	0.548	^*^	0.305		0.365	
Soft drinks and sugared fruit drinks	−0.287		−0.220		−0.340		−0.307	
**Activity preferences**								
**Physical activities**								
Walking, Tai Chi	0.395	^*^	0.187		0.304		0.218	
Body building	−0.002		−0.230		0.051		0.131	
Sports (ping pong, badminton, tennis, soccer, basketball, volleyball)	0.520		0.002		0.349		0.363	
**Sedentary activities**								
Reading	−0.174		0.433	^**^	−0.162		−0.565	
Watching TV	0.153		0.416	^***^	0.519	^*^	0.476	^*^
Playing computer/video games, surfing the internet	−0.223		−0.106		0.119		0.346	
**Interaction effect**								
Reading × highest educational level							0.374	^*^

Sex and highest educational level are taken as control variables in above models.

^*^p < 0.05; ^**^p < 0.01; ^***^p < 0.001.

## 4. Discussion

Positive attitudes toward life in general may be especially important in old age (29). Longevity is an indicator of active healthy aging, but the subjective wellbeing of older adults is an important factor in measuring successful healthy aging (30). Optimistic attitudes and subjective wellbeing in the daily life of older adults had a strong protective effect on all-cause mortality (31). The present study elucidated that subjective wellbeing was a protective factor for mortality in rural older adults. Subjective wellbeing above or equal to the average level decreased the death risk of rural elderly people by 10%, and subjective wellbeing above or equal to the sum of the average level and one standard deviation decreased the risk of death by 69%. These results suggest that drastically improving the subjective wellbeing of rural elderly people would decrease the number of deaths.

The subjective wellbeing of rural elderly people of different birth generations first decreased then increased slightly. This result may reflect the fluctuation of subjective wellbeing caused by the policy effect. Specifically, the subjective wellbeing of rural elderly people from 2006 to 2009 presented a downward trend in all three birth generations. The subjective wellbeing of rural elderly people from 2009 to 2011 and the declining trend of the 1940s and 1930s birth generations were slower than rural elderly people from 2006 to 2009, but the 1908–1920s birth generation showed an upward trend. As a transition point, this result may be because the Chinese government began to make special investments in the elderly care service system in 2009 and focused on supporting the construction of public elderly care service institutions and township nursing homes. The pilot reform of the new rural social endowment insurance for rural residents began in 2009, and social insurance for rural farmers was initiated. Research has detected that Chinese rural farmer who participated in the New Rural Cooperative Medical System enhanced their subjective wellbeing (32). Analogously, Medicare creates a safety net for vulnerable populations in the United States, which ensures a greater sense of economic and health security for older people and their families (33).

The trend of subjective wellbeing from 2011 to 2015 revealed that these trends for different birth generations are all on the rise. The changing trajectory of the subjective wellbeing of elderly people in rural China with age is likely to benefit from the continuous improvement of the Chinese elderly welfare system, such as China's Undertakings for the Aged during the 12th Five-year Plan (2011–2015), and the social elderly care service system construction plan (2011–2015). Previous studies demonstrated that social age-friendly policies were conducive to increased wellbeing of elderly people, such as the pension policy in Korea (34), social income policy in Turkey (35), internet access policy in South Africa (36), age-friendly city strategy in China (37), and ongoing construction of Chinese happy villages. We should pay attention to popularizing the policy needs of elderly people and their families and improve the policy literacy of this group. Notably, when seeking practical and policy interventions for the social welfare of elderly people, the characteristics of elderly people of different birth generations and age stages should be considered.

Lifestyle is an important factor affecting happiness (38). For elderly people, the most commonly discussed lifestyle issues are diet and activity. Food is a necessity of life that has an important impact on mental health (39). Research has found that the eating behaviors and food preferences of adults predict their wellbeing (40). We elucidated the positive effect of a preference for eating vegetables on the subjective wellbeing of rural elderly people born in the 1940s and 1930s, but it had no significant effect on rural elderly people born in the 1908–1920s. This difference may be because rural elderly people of earlier birth generations have experienced resource poverty since childhood and have no specific dietary preferences, and dietary habits do not affect their subjective wellbeing. Preferences for eating fruits and drinking soft drinks and sugared fruit drinks had no significant effect on the subjective wellbeing of any birth generation in the rural elderly population. In contrast to vegetables on the dining table, fruits and drinks are not necessary for most rural elderly people and are considered additional consumption.

Leisure time physical activities improve wellbeing and alleviate depression and anxiety among elderly people (41–43). Our study demonstrated that the positive effect of walking/Tai Chi on subjective wellbeing was significant among rural elderly people born in the 1940s, but it was not significant in the other two birth generations. Younger rural elderly people may have had better activities of daily life abilities than older rural elderly people and paid more attention to physical exercise. Physical activity maintains the mobility and physical functions of elderly people and improves their community and social engagement (44, 45). We encourage rural elderly people with the necessary ability to participate in regular physical exercise of appropriate intensity, such as walking, performing Tai Chi, doing housework and aerobic exercise. Tai Chi is one of the most popular and frequently practiced sports among older Chinese people (46). For rural elderly people with poor activity ability, simple home-based health exercises may be performed according to their own physical conditions.

We observed that the positive effect of the preference for watching TV on subjective wellbeing was not significant in rural elderly people born in the 1940s, but it was significant in rural elderly people born in the 1930s and 1908–1920s. Barriers to participation in activities, such as sports, tend to increase with age (47). Senior rural elderly people tend to choose more sedentary activities, and watching TV is an option. Significantly, people experience abundant subjective wellbeing when they are engaged in interesting activities (48). We found that rural elderly people born in the 1930s who liked reading had a higher sense of subjective wellbeing than rural elderly people who do not like reading. A British survey found that leisure readers reported less stress and depression on average than non-readers (49). For elderly people, reading is a relatively relaxed leisure activity that promotes emotional health and brings a stronger sense of relaxation than other leisure activities (50). In addition to visual and performing arts activities, we advocate that communities perform reading activities in various forms, including reading clubs, essay competitions, and the appreciation of classical works. Providing opportunities for the community participation of elderly people in multiple dimensions reduces their social isolation and loneliness and improves their subjective wellbeing.

A major strength of the present study is that it was based on a large nationally representative sample, and we examined the longevity and wellbeing of rural elderly people in whom we were interested. The prospective study design of CHNS helped eliminate the potential recall bias that is an issue of special concern in most retrospective *post-hoc* analyses. The limitations of this study also merit consideration. First, the sample size of the rural elderly people involved in this study was relatively small. However, CHNS adopts a multistage, random clustered sampling process, the study participants were representative, and the results may be used as a reference for policy-making. Second, our sample was Chinese rural elderly people, and possible differences between Eastern and Western cultures should be considered when extrapolating the research results to other countries. Third, the choice of covariates was not sufficiently comprehensive. There are many factors associated with longevity and subjective wellbeing, such as health status for longevity and income for subjective wellbeing. However, this study focused on the perspective of food and activity preferences. Due to the few rural elderly people who answered the measures of present medical illness and income in the CHNS from 2006 to 2015, this study did not include this them in the model control, which is also a limitation of this study. Finally, CHNS is aimed at all age groups, rather than just the elderly, and the categories of activity preference choices may not be representative for the rural elderly.

## 5. Conclusion

The present study contributes additional evidence that improving subjective wellbeing extends life span and reduces mortality risk among rural elderly people. Improving the subjective wellbeing of rural elderly people may be achieved by shaping a healthy lifestyle, such as a preference for eating vegetables, walking/Tai Chi, and reading. We found that a higher educational level of rural elderly people and a greater enjoyment of reading were associated with higher subjective wellbeing. It is recommended that the implementation of facility, infrastructure, space and equipment be strengthened to promote wellbeing in rural regions.

## Data availability statement

The datasets used and/or analyzed during the current study are available from the corresponding author on reasonable request.

## Ethics statement

CHNS was approved by the Institutional Review Boards of the University of North Carolina at Chapel Hill and the Chinese Center for Disease Control and Prevention. The patients/participants provided their written informed consent to participate in this study.

## Author contributions

XL and Y-CC made contributions to data analysis and interpretation. XL, MG, SH, and ZF drafted the manuscript. XL, MC, TC, C-YL, and Y-CC revised it critically for important intellectual content. C-YL and Y-CC supervised the study. TC and Y-CC provides funding acquisition. All authors were major contributors in conception and design of the study, read, and approved the final manuscript. All authors agree to be accountable for all aspects of the work in ensuring that questions related to the accuracy or integrity of any part of the work are appropriately investigated and resolved.
